# Causative Species and Serotypes of Shigellosis in Mainland China: Systematic Review and Meta-Analysis

**DOI:** 10.1371/journal.pone.0052515

**Published:** 2012-12-20

**Authors:** Zhili Chang, Shuting Lu, Lihong Chen, Qi Jin, Jian Yang

**Affiliations:** 1 MOH Key Laboratory of Systems Biology of Pathogens, Institute of Pathogen Biology, Chinese Academy of Medical Sciences & Peking Union Medical College, Beijing, China; 2 Institute of Medical Information/Medical Library, Chinese Academy of Medical Sciences & Peking Union Medical College, Beijing, China; Rockefeller University, United States of America

## Abstract

**Background:**

*Shigella,* the causative agent of shigellosis, is a major global public health concern, particularly in developing countries with poor sanitation. A comprehensive and current understanding of the prevalent species and serotypes of shigellosis is essential for both disease prevention and vaccine development. However, no current data are available on the causative species/serotypes of shigellosis in mainland China during the past decade.

**Methods and Findings:**

Relevant studies addressing the prevalent species of shigellosis in mainland China from January 2001 to December 2010 were identified from PubMed and the Chinese BioMedical Literature Database (in Chinese) until April 2012. A total of 131 eligible articles (136 studies) were included in this review. Meta-analyses showed that the prevalences of *S. flexneri* and *S. sonnei* were 76.2% (95% CI, 73.7%–78.5%) and 21.3% (95% CI, 19.0%–23.7%), respectively. Stratified analyses indicated a decrease in the prevalence of *S. flexneri* cases and an increase in the prevalence of *S. sonnei* cases concurrent with the rapid economic growth experienced by China in recent years. Moreover, significantly higher rates of *S. sonnei* were observed in the East, North and Northeast regions of China, as compared to the rest of the country. These phenomena imply the possible association between the prevalent species of *Shigella* and regional economic status; however, additional factors also exist and require further investigations. Moreover, the two major serotypes *S. flexneri* 2a and 4c accounted for 21.5% (95% CI, 16.7%–27.4%) and 12.9% (95% CI 9.8%–16.9%) of *S. flexneri* infections, respectively, in the past decade. However, these results were found to be frequently heterogeneous (p for Q tests <0.01).

**Conclusions:**

This study provides an updated review of the causative agents of shigellosis in mainland China and focuses on the importance of strengthening prevention and research efforts on *S. sonnei* and the newly emerged *S. flexneri* serotype 4c.

## Introduction

*Shigella*, a group of Gram-negative, non-spore forming and rod-shaped bacteria, are the causative agents of shigellosis (or bacillary dysentery). The bacteria are considered to be highly contagious because of their low infectious inoculum size (10–100 organisms) [1]. *Shigella* is primarily transmitted through the fecal-oral route; therefore, it is still a major global public health threat, particularly in developing countries with poor sanitation conditions. One hundred sixty-five million episodes of shigellosis are estimated to occur worldwide annually, among which 163 million occur in resource-poor countries [2]. Moreover, a recent surveillance study in six Asian countries suggested that the global burden of *Shigella* infection might be much higher than these estimates [3]. Nonetheless, the number of *Shigella*-related deaths in Asia has substantially decreased due to current nonspecific interventions, including measles vaccination, vitamin A supplementation, and improved nutrition [4]. However, the treatment of shigellosis has recently become more difficult due to the emergence of multidrug-resistant strains, which has narrowed the clinical choice of antimicrobial agents [3].

*Shigella* bacteria are serologically grouped into four species (*S. dysenteriae*, *S. flexneri*, *S. boydii*, and *S. sonnei*), which are further subdivided into more than 40 serotypes based on the O-antigen structures of the membrane-associated lipopolysaccharide [5]. Though these species share similar pathogenic properties, they exhibit unique epidemiological characteristics. For instance, *S. flexneri* is known to be predominant in developing countries (median 60%), while *S. sonnei* is the most common species found in the industrialized world (median 77%) [2]. However, recent studies have indicated that *S. sonnei* is overtaking *S. flexneri* in newly industrialized nations such as Thailand, South Korea and Taiwan [3], [6], [7]. As initial *Shigella* infection produces only homologous, not heterologous, serotype protection [8], a comprehensive and current understanding of the prevalent *Shigella* species and serotypes is essential for the development of an effective vaccine [9]. Currently, though several strategies have been used to develop vaccines targeting shigellosis, only one live bivalent *S. flexneri* 2a and *S. sonnei* vaccine is licensed in China [8], [10]. Updated information on the circulating strains of *Shigella* is also important to enable assessment of the impacts of vaccination.

A retrospective review indicated that *S. flexneri* was responsible for 86% of shigellosis episodes in China during 1991–2000, and the predominant serotype was identified as *S. flexneri* 2a (80%) [11]. However, concurrent with the rapid economic development in China during the past decade, both morbidity and mortality from shigellosis have decreased steadily every year, based on annual reports from China’s Center for Disease Control and Prevention. A recent study using the national surveillance data from 2009 showed that the annual shigellosis morbidity rate was 20.28 cases per 100,000 people in mainland China, with *S. flexneri* (67.3%) and *S. sonnei* (32.7%) as the two major causative species [12]. Nevertheless, no comprehensive data are currently available regarding the causative species and serotypes of shigellosis in mainland China during the past decade. In this study, we performed a systematic review and meta-analysis of the most recently published data on *Shigella* species and serotypes circulating in mainland China during 2001–2010.

## Methods

### Data Sources and Search Strategy

We systematically searched PubMed/Medline (in English) and the Chinese BioMedical Literature Database (in Chinese) for articles published between January 2001 and April 2012. Various combinations of the terms “*Shigella*”, “shigellosis”, “bacillary dysentery” and “China” were used to identify potentially relevant studies.

### Inclusion and Exclusion Criteria

Publications addressing the prevalent species of shigellosis in mainland China during January 2001 to December 2010 were thought to be relevant. The primary inclusion criterion was that the studies should report precise isolate numbers of the different *Shigella* species. In addition, the identification of *Shigella* species/serotypes should be based on the results of both biochemical reactions according to standard methods and serological approaches confirmed by slide agglutination test using commercially available antisera. If the study was reported in duplicate, the article with the earlier publication date was included. Review articles, congress abstracts, studies reported in languages other than English or Chinese, data from regions of China other than mainland (i.e. Taiwan and Hong Kong), study duration before January 2001, studies limited to outbreaks, or studies containing discrepant data were excluded. To minimize any potential sample size bias, articles presenting fewer than 50 *Shigella* isolates were excluded.

### Data Abstraction

Two reviewers (ZC and SL) independently extracted data from the included studies. We gathered the following information from original publications: first author and year of publication, province of the study investigated, medical treatment type, study duration, total sample size, individual number of each species of *Shigella* isolate, and individual number of each serotype for *S. flexneri* isolates (if any). Discrepancies in either the decision regarding inclusion/exclusion of studies or regarding the data extracted were resolved by discussion with a third reviewer (JY).

### Available of National and Regional Economic Data

Annual data for the gross domestic product (GDP) per capita of China and the gross regional product (GRP) per capita of each province for each year between 2001 and 2010 were retrieved by querying the “national accounts” subject in the China Statistical Database from the National Bureau of Statistics of China (http://www.stats.gov.cn/). The decade averages calculated from the annual data for both GDP and GRP per capita were used as indicators of economic status for mainland China and each province.

### Statistical Analysis

The chi-square test was used to estimate differences in the prevalent *Shigella* species in different groups where appropriate. Between-study heterogeneity across all eligible comparisons was estimated by the Cochran Q test (P<0.01 was considered to be indicative of statistically significant heterogeneity) and the I^2^ statistic (values of 25%, 50%, and 75% represent low, medium, and high heterogeneity, respectively). DerSimonian-Laird random-effects models were used to calculate summary estimates when significant heterogeneity among studies was detected; otherwise, fixed-effects models were applied. All meta-analyses were performed individually using the Comprehensive Meta-Analysis software (Version 2.0, Biostat, Englewood, NJ, USA). Stratified analyses were subsequently performed according to the geographic regions in China (East, North, Middle, South, Northwest, Southwest and Northeast), the GRP per capita (top 10 provinces and the others), two modern cities in mainland China (Beijing and Shanghai), the study period (2001–2005 and 2006–2010), and the medical treatment type (clinic and hospital), respectively.

## Results

**Figure 1:**
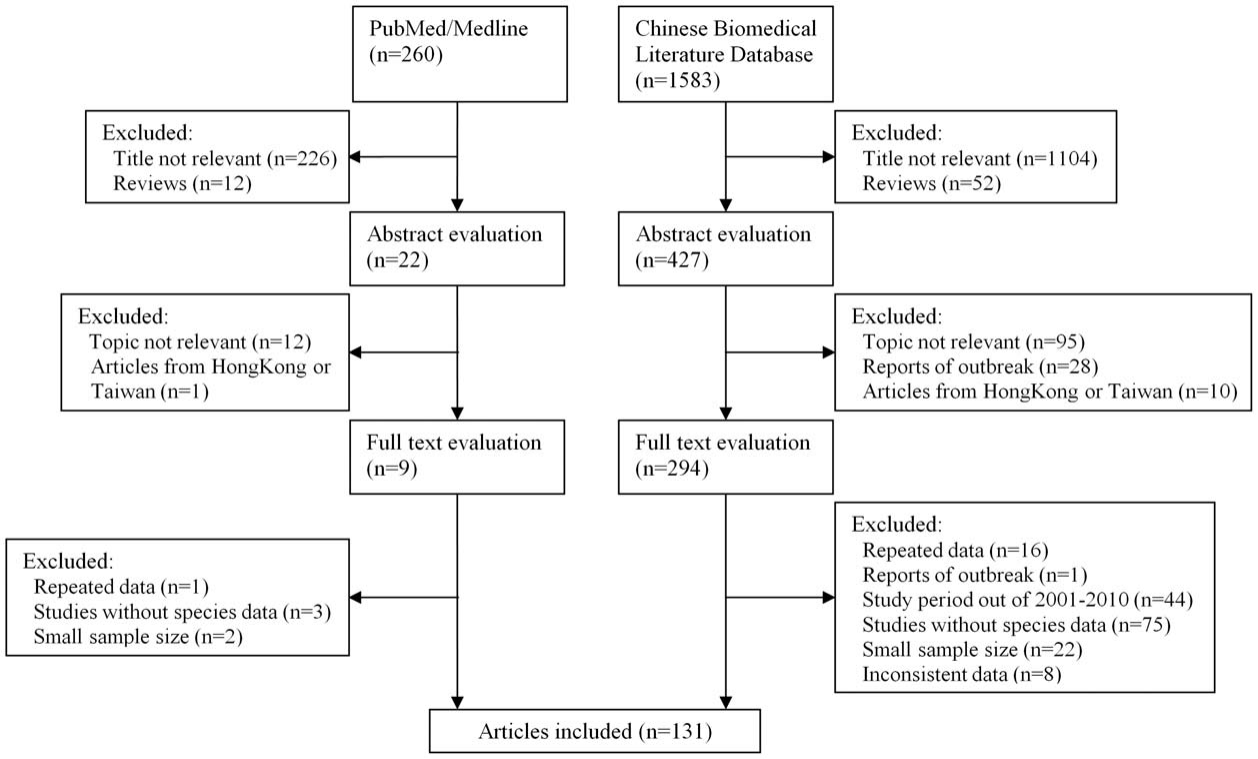
Flow diagram of study identification.

We identified a total of 1,843 articles by literature search using different combinations of key terms from the databases, as shown in **Figure 1**. After exclusion based on title and abstract evaluation, 303 articles (9 in English and 294 in Chinese) were retrieved for detailed full-text review. Of these, 172 were further excluded according to the exclusion criteria. Detailed information on study identification is available in **Table S1**. In total, 131 articles (136 studies) were analyzed for the prevalence of *Shigella* species and/or *S. flexneri* circulating serotypes. **Figure 1** shows the detailed selection process and the number of articles included/excluded in each phase.

**Figure 2:**
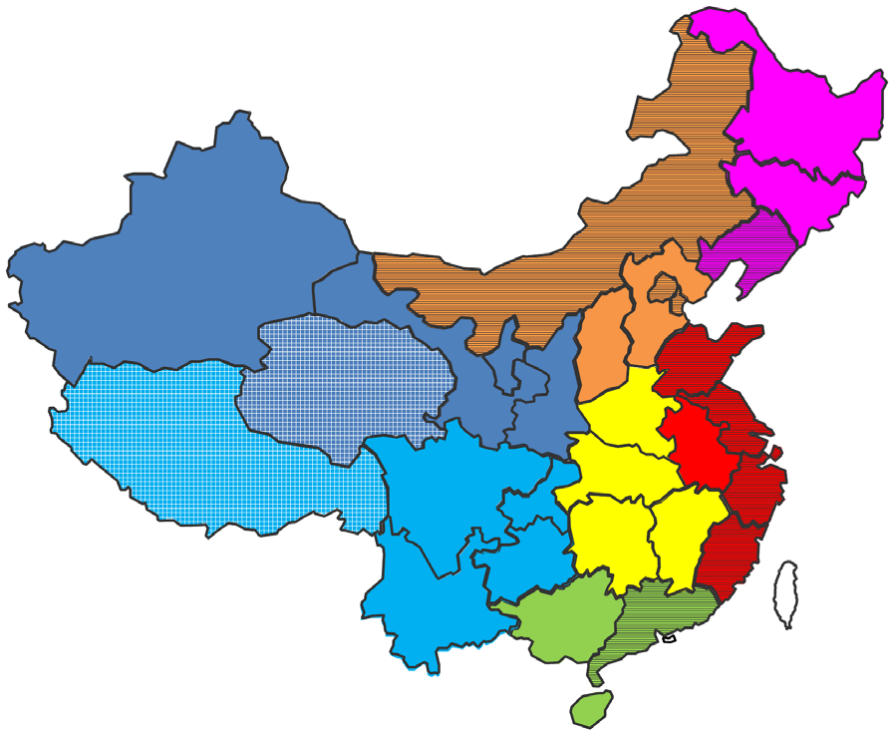
Geographic demarcation of mainland China, color coded as follows: red, East; orange, North; yellow, Middle; green, South; blue, Northwest; cyan, Southwest; violet, Northeast. Two provinces without available data are marked with light grids. The top 10 economic provinces are highlighted by stripes.

As shown in **Figure 2** and **Table S2**, the included 136 studies were conducted in 29 of 31 provinces in mainland China. However, only 63 studies from 19 provinces provided detailed information on the prevalent *S. flexneri* serotypes (**Table S2**). Sample sizes of the studies ranged from 50 to 1,466, and a total of 30,959 *Shigella* isolates were analyzed in the 136 studies. More studies were performed in East (61) and North (24) China, as compared to the other geographic regions. The number of studies from Zhejiang, Shanghai and Beijing were 20, 19 and 15, respectively, whereas only one study was available for each of the other eight provinces (**Table S2**). In addition, 61.8% of the studies (84/136) were conducted in the 10 provinces with an average GRP per capita that was higher than the national GDP per capita, during 2001–2010 (abbreviated as “top 10 economic provinces” thereafter).

The meta-analyses of the prevalence of *Shigella* species circulating throughout mainland China during the past decade are shown in **Table 1**. *S. flexneri* and *S. sonnei* were found to be the two predominant species, with summarized prevalences of 76.2% (95% CI, 73.7%–78.5%) and 21.3% (95% CI, 19.0%–23.7%), respectively. However, evident heterogeneity was observed for both species. Moreover, the prevalence of the two species showed clear diversity in stratified analyses by geographic area, regional economic status, study period, and medical treatment type. For example, *S. flexneri* and *S. sonnei* individually accounted for 64.6% (95% CI, 57.2%–71.4%) and 34.9% (95% CI, 28.2%–42.3%) of shigellosis cases in North China, respectively, but the proportions were 85.9% (95% CI, 79.4%–90.6%) and 9.4% (95% CI, 5.8%–15.0%), respectively, in Northwest China. In general, a lower rate of *S. flexneri* cases and a higher rate of *S. sonnei* cases were observed in studies from East, North and Northeast China, as compared to the other regions (**Table 1**). In addition, a similar proportion of differences in the prevalent *Shigella* species also existed for the top 10 economic provinces, as compared to the other 21 provinces in mainland China. Interestingly, though Beijing and Shanghai are the two biggest modern cities in China and have very similar economic statuses, significant differences in the proportions of dominant *Shigella* species were observed (p<0.001); *S. flexneri* accounted for only 56.0% of shigellosis cases in Beijing and 70.2% in Shanghai, whereas *S. sonnei* accounted for 43.3% of shigellosis cases in Beijing and only 29.6% in Shanghai (**Table 1**). Moreover, the prevalence of *S. flexneri* in 2006–2010 was significantly lower than that in 2001–2005; in contrast, the situation for *S. sonnei* was just the opposite (p<0.001 for both). Similarly, *S. flexneri* infections constituted 67.1% of shigellosis cases in outpatients and 88.4% in inpatients, whereas *S. sonnei* infections constituted 29.9% of shigellosis cases in outpatients and only 7.4% in inpatients (**Table 1**). **Figure S1** shows forest plots of meta-analyses on the prevalence of *S. flexneri* and *S. sonnei*.

**Table 1:** Prevalence of causative species of shigellosis in mainland China, 2001–2010.

			*S. flexneri*				*S. sonnei*			
	No. of studies	No. of isolates	Prevalence (95% CI) (%)	n	Heterogeneity test		Prevalence (95% CI) (%)	n	Heterogeneity test	
					I^2^ (%)	P			I^2^ (%)	P
**Total**	136	30959	76.2(73.7–78.5)	22973	95.5	0	21.3(19.0–23.7)	7260	95.6	0
**Stratified by geographic area**										
East	61	13747	74.1(70.8–77.1)	10251	93.6	0	23.7(20.8–26.9)	3316	93.7	0
North	24	5962	64.6(57.2–71.4)	3723	96.2	0	34.9(28.2–42.3)	2196	96.1	0
Middle	15	2831	83.5(75.3–89.4)	2315	95.5	0	14.6(10.0–21.0)	444	93.3	0
South	8	1049	82.5(74.4–88.4)	819	87.7	0	15.3(9.9–22.8)	201	86.1	0
Northwest	17	5395	85.9(79.4–90.6)	4401	97.1	0	9.4(5.8–15.0)	709	97.0	0
Southwest	6	911	80.6(57.6–92.8)	740	95.4	0	17.5(5.8–42.2)	148	95.9	0
Northeast	5	1064	76.2(62.7–85.9)	724	92.0	0	22.3(12.6–36.3)	333	92.7	0
**Stratified by regional economy**										
GRP top 10	84	19666	71.8(68.6–74.8)	13862	95.2	0	26.3(23.4–29.5)	5606	95.3	0
Others	52	11293	82.7(78.9–85.9)	9111	95.4	0	14.4(11.5–17.7)	1741	94.7	0
**Stratified by modern city**										
Beijing	15	4657	56.0(47.8–63.8)	2666	95.6	0	43.3(35.5–51.5)	1950	95.7	0
Shanghai	19	5891	70.2(65.0–74.9)	4248	93.3	0	29.6(24.9–34.8)	1635	93.2	0
**Stratified by study period**										
2001–2005	77	16706	82.0(79.3–84.4)	13390	93.8	0	15.7(13.3–18.3)	3013	94.2	0
2006–2010	56	9386	67.3(62.1–72.1)	6019	95.6	0	29.9(25.3–35.0)	3133	95.6	0
**Stratified by medical treatment type**										
Clinic	45	10801	67.1(62.2–71.7)	7302	93.8	0	29.9(25.2–35.1)	3242	96.2	0
Hospital	7	1082	88.4(78.7–94.0)	938	95.6	0	7.4(3.0–17.0)	107	92.6	0

Abbreviations: GRP, gross regional product; n, number of events (isolates of *S. flexneri* or *S. sonnei*).

*S. flexneri* is subdivided into 15 serotypes, whereas *S. sonnei* is clonal. Therefore, we further investigated the distribution of the causative serotypes of *S. flexneri* episodes (**Table 2**). *S. flexneri* 2a (21.5%, 95% CI, 16.7%–27.4%) was still the predominant serotype among *S. flexneri,* followed by *S. flexneri* 4c (12.9%, 95% CI 9.8%–16.9%), *S. flexneri* 1a (11.2%, 95% CI, 9.4%–13.3%), *S. flexneri* 2b (7.6%, 95% CI, 5.9%–9.7%) and *S. flexneri* x (4.7%, 95% CI, 3.4%–6.4%). However, high heterogeneity was observed in the results of most serotypes. For example, *S. flexneri* 2a presented as the dominant serotype in only 21 of the 63 studies included. Instead, *S. flexneri* 4c was the found to replace *S. flexneri* 2a as the predominant serotype in 19 studies. Furthermore, *S. flexneri* 4c was completely not detected in more than half of the included studies (**Table S2**).

**Table 2:** Distribution of *S. flexneri* serotypes in mainland China, 2001–2010 (63 studies included).

S. flexneri serotype	No. of isolates	Prevalence (95% CI) (%)	Heterogeneity test	
			I^2^ (%)	P
**1**	71	0.8(0.5–1.4)	81.0	0
**1a**	1283	11.2(9.4–13.3)	85.8	0
**1b**	102	1.1(0.8–1.7)	71.8	0
**2**	251	1.1(0.6–2.0)	92.5	0
**2a**	2781	21.5(16.7–27.4)	96.8	0
**2b**	1069	7.6(5.9–9.7)	91.0	0
**3a**	82	1.0(0.6–1.5)	69.2	0
**3b**	9	0.6(0.4–0.8)	0.0	0.99
**4**	781	2.8(1.8–4.5)	94.5	0
**4a**	253	1.6(1.0–2.5)	89.5	0
**4b**	67	0.9(0.6–1.4)	63.8	0
**4c**	2128	12.9(9.8–16.9)	94.3	0
**5**	11	0.6(0.5–0.9)	10.0	0.26
**5a**	16	0.6(0.5–0.8)	0.0	0.99
**5b**	119	0.9(0.6–1.3)	75.0	0
**6**	133	0.9(0.5–1.6)	87.4	0
**X**	773	4.7(3.4–6.4)	91.7	0
**Y**	165	1.8(1.4–2.4)	50.3	0
**Others** *	114	1.4(1.0–1.9)	55.4	0
**Total**	10208	–	–	–

* Including untyped cases.

## Discussion

This review addressed the status of the prevalent species and serotypes of shigellosis in mainland China during 2001–2010. One hundred thirty-one relevant articles, 3 in English and 128 in Chinese, were identified and summarized by meta-analysis. Our estimates showed that *S. flexneri* (76.2%, 95% CI, 73.7%–78.5%) and *S. sonnei* (21.3%, 95% CI, 19.0%–23.7%) were the two major causative species of *Shigella* circulating in mainland China during the past decade. Our results are generally in agreement with previous estimates from the national surveillance data from 2009 [12].

Mainland China consists of 31 provinces with over 1 billion people living in diverse areas with varying geographies, environments, economies, customs, and even climates. Therefore, geographic heterogeneity can be expected in the proportions of the aforementioned two major *Shigella* species. Indeed, significantly higher rates of *S. sonnei* cases and lower rates of *S. flexneri* cases were observed in East, North and Northeast China, as compared to those in Middle, South, Northwest and Southwest China (**Table 1**). Using the decade average of GRP per capita as an indicator, we ranked the economic status of each province in mainland China during the past decade (data not shown). Similar to the geographic differences discussed above, significantly higher rates of *S. sonnei* cases and lower rates of *S. flexneri* cases were also observed in the top 10 economic provinces, as compared to those in the other 21 provinces (**Table 1**). Furthermore, these results are internally consistent given that among the top 10 economic provinces in China, five are from the East, three are from the North, and one is from the Northeast, whereas only one province is from South China (**Figure 2**). Hence, the observed geographic differences in the proportions of the two causative *Shigella* species in mainland China are likely associated with the overall economic status of the different regions.

Historically, *S. flexneri* is known to be predominant in developing countries. In this study we observed that more than 70% of shigellosis was caused by *S. flexneri* in most geographic regions of mainland China (**Table 1**). Interestingly, the overall proportion of *S. flexneri* decreased by ∼10%, whereas that of *S. sonnei* increased by ∼10% during the past decade, as compared to the data from 10 years ago (1991–2000) [11]. Nevertheless, the previous estimates were produced from a systematic review rather than a meta-analysis as performed in this study; thus, direct comparison of the two values might not be valid. Our stratified analysis based on study duration indicates the same trends of causative species proportion changes of shigellosis in the past decade: *S. flexneri* cases decreased from 82.0% (95% CI, 79.3%–84.4%) in 2001–2005 to 67.3% (95% CI, 62.1%–72.1%) in 2006–2010, while *S. sonnei* cases increased from 15.7% (95% CI, 13.3%–18.3%) in 2001–2005 to 29.9% (95% CI, 25.3%–35.0%) in 2006–2010. Therefore, an undeniable tendency for *S. flexneri* cases to decease and *S. sonnei* cases to increase over time persisted in mainland China during the past decade. Cases of *S. sonnei* are also reported to have surpassed *S. flexneri* in newly industrialized countries, such as Thailand, South Korea and Taiwan [3], [6], [7]. Therefore, the observed causative species transition of shigellosis in mainland China might be linked to improvements in economic level, considering that China has experienced rapid economic growth during the past decade.

It is noteworthy that both geographic and temporal analyses implied that the prevalent species of *Shigella* are well associated with regional economic status. However, significant differences in the proportions of *S. flexneri* and *S. sonnei* were observed between Beijing and Shanghai, two modern cities with similar economic levels. These findings suggest that additional factors are likely contributing to the proportions of predominant *Shigella* species. Nonetheless, the essential factors that are driving the phenomenon are not yet known. Determination of the key factor(s) that influence the regional dominant *Shigella* species is critical for the development of better global shigellosis control and prevention practices in the future. As the *Shigella* species transition trend currently observed in China is likely to remain in the near future, the current and follow-up statuses of the populations of the bacteria, as well as their hosts in China, might be good targets for further investigations.

*S. flexneri* serotype 2a is known to have been responsible for 50–70% of endemic and pandemic shigellosis in mainland China in the last century [13]. Previous review also revealed that serotype 2a accounted for 80% of *S. flexneri* episodes during 1991–2000 [11]. However, our results indicate that *S. flexneri* 2a remained the dominant serotype in only one third of the studies, and the overall proportion has dramatically decreased to about 20% during the past decade. The decrease of *S. flexneri* 2a cases might be associated with the bivalent *S. flexneri* 2a and *S. sonnei* vaccine licensed in China in 1997 [8], [10], as the vaccination campaign may represent a new selective pressure on the pathogen [14]. Nevertheless, the current *Shigella* vaccine in China is an optional rather than obligatory vaccine in the national immunization program, and the information on vaccination rates is unavailable publicly. Therefore, further evaluations of the impact of the vaccine on *Shigella* serotype replacement in China will be an interesting focus in future studies.

Notably, the newly emerged *S. flexneri* 4c overtook *S. flexneri* 2a as the predominant serotype in approximately one third of the studies. *S. flexneri* 4c was first identified in Romania in the 1960s and was observed to differ biochemically from *S. flexneri* 4a and 4b representatives [15]. Moreover, *S. flexneri* 4c was found to account for as much as 17.2% of all *Shigella* isolates in the former Union of Soviet Socialist Republics (USSR) in the 1980s [15]. A recent study reported that fluoroquinolone-resistant *S. flexneri* 4c clones were isolated from East China [16]. So further investigations on *S. flexneri* serotype 4c circulated in China are urgently required. In addition, shigellosis cases caused by several other serotypes of *S. flexneri*, including serotypes 1a (11.2%), 2b (7.6%) and x (4.7%), are also significantly increased during the past decade as compared with previous results [11]. Therefore, to achieve better immune protections in China these additional causative *S. flexneri* serotypes should be incorporated into further *Shigella* vaccine developments.

The etiology distribution variation in each medical treatment type (clinic and hospital) may serve as a surrogate of disease severity of different *Shigella* species. Our results showed that the prevalence of *S. flexneri* in inpatients was significantly higher than that in outpatients; in contrast, the situation for *S. sonnei* was just the opposite (p<0.001 for both) (**Table 1**). This is in good agreement with the previous genetic results that *S. sonnei* is generally less virulent than *S. flexneri* from the genomic viewpoint [17]. Nevertheless, the threats from *S. sonnei* in mainland China should not be underestimated. Among the 29 studies addressing outbreaks that were excluded from the meta-analysis, 27 studies performed serological analyses, and *S. flexneri* and *S. sonnei* were responsible for 16 (59.3%) and 11 (40.7%) of the outbreaks, respectively. In addition, instead of a single or several predominant serotypes as might be expected, eight different serotypes of *S. flexneri* were confirmed to be associated with the 11 outbreaks that done detailed serotyping (data not shown). The results further highlight the aforementioned necessity to improve the valency coverage of currently conceived *Shigella* vaccines.

A recent multicenter study in six Asian countries found that a surprising 92% of shigellosis in China was caused by *S. flexneri* [3]. However, the surveillance was conducted in a rural area of China in 2002, which is far from representative of the current situation of *Shigella* prevalence in mainland China. The meta-analysis presented here combines data across studies to estimate the most current status of *Shigella* infections in China with more precision than is possible in a single study. Nevertheless, some limitations of this review should be considered. First, our results may not be fully representative of mainland China. The species information was not available for two provinces, both of which are in west of China (**Figure 2**). In addition, *S. flexneri* serotype information was not available, or did not meet the inclusion criteria, for 12 provinces in this analysis. Even for the provinces included by this study, evident discord was observed in a number of studies included for each province. However, we tried to ensure that the most valid studies were included through restrictive inclusion criteria. Second, several helpful stratify analyses were restricted by the limited information available from the original publications. For example, the primary targets of *Shigella* vaccines being developed are children under 5 years old, so the epidemiology of species/serotypes by meaningful age category would be very informative. However, only 15 of the 136 studies provided information on age of patients, and these studies employed different grouping criteria, which makes the stratify analysis using the basic age category can only include three studies (**Table S2**). Such data is obviously far from representing meaningful results. Third, several potential biases should be kept in mind for results interpretation. Only 3 of the 136 studies included were population-based; thus, the selection of subjects might have made the results prone to potential selection bias. Though the species/serotypes identification in all studies included was confirmed by both biochemical reactions and serological results, the sensitivity and coverage of the antisera sets used by each study may variant, which might have biased the results. The commercial *Shigella* antisera are usually available in different sets that offer diverse coverage of species/serotypes to accommodate the requirements of different users. But the details of the antisera set used in each study is generally absent from the publications, leading to the exclusion or evaluation of the potential bias impossible. In addition, historical improvements of the sensitivity in serological testing might have led to more accurate serotyping in recent studies than in previous work. Forth, the antimicrobial resistance patterns of *Shigella* isolates could not be analyzed due to the limited information available from the original publications.

To our knowledge, this is the first study to systematically review the status of circulating strains of *Shigella* in mainland China during the past decade. Our results reveal significant changes in the causative species and serotypes of shigellosis in recent years and emphasize the necessity to focus more attention on *S. sonnei* and *S. flexneri* serotype 4c episodes in future shigellosis prevention and control measures, as well as in basic research. In addition, our study highlights the limited information about *Shigella* epidemiology in many provinces of China with relatively low economic status. To achieve a precise estimate, further large-scale population-based surveillance studies using standardized methods are required.

## Supporting Information

Figure S1 **Forest plots of meta-analyses on the prevalence of** ***S. flexneri*** **(A) and** ***S. sonnei*** **(B).**
(PDF)

Table S1 **Included and excluded articles in full-text evaluation.**
(DOC)

Table S2 **Detailed information of the included studies.**
(XLS)
