# Supplementary figures and images for: Causative Species and Serotypes of Shigellosis in Mainland China: Systematic Review and Meta-Analysis
Source: PLoS One. 2012 Dec 20;7(12):e52515. doi: 10.1371/journal.pone.0052515 (PMC3527545; doi:10.1371/journal.pone.0052515)

# Prevalence of Shigella flexneri

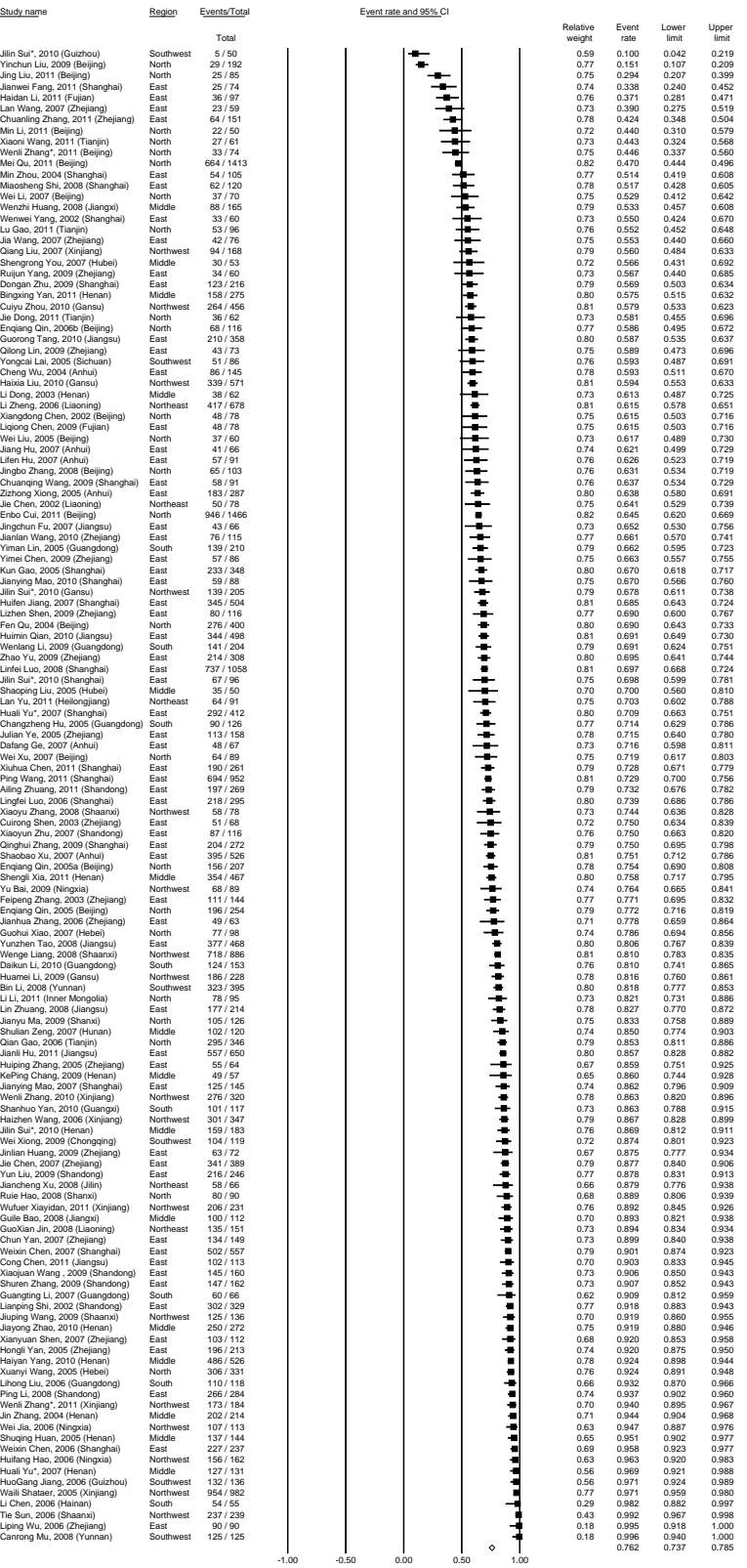

Meta Analysis

# B Prevalence of Shigella sonnei

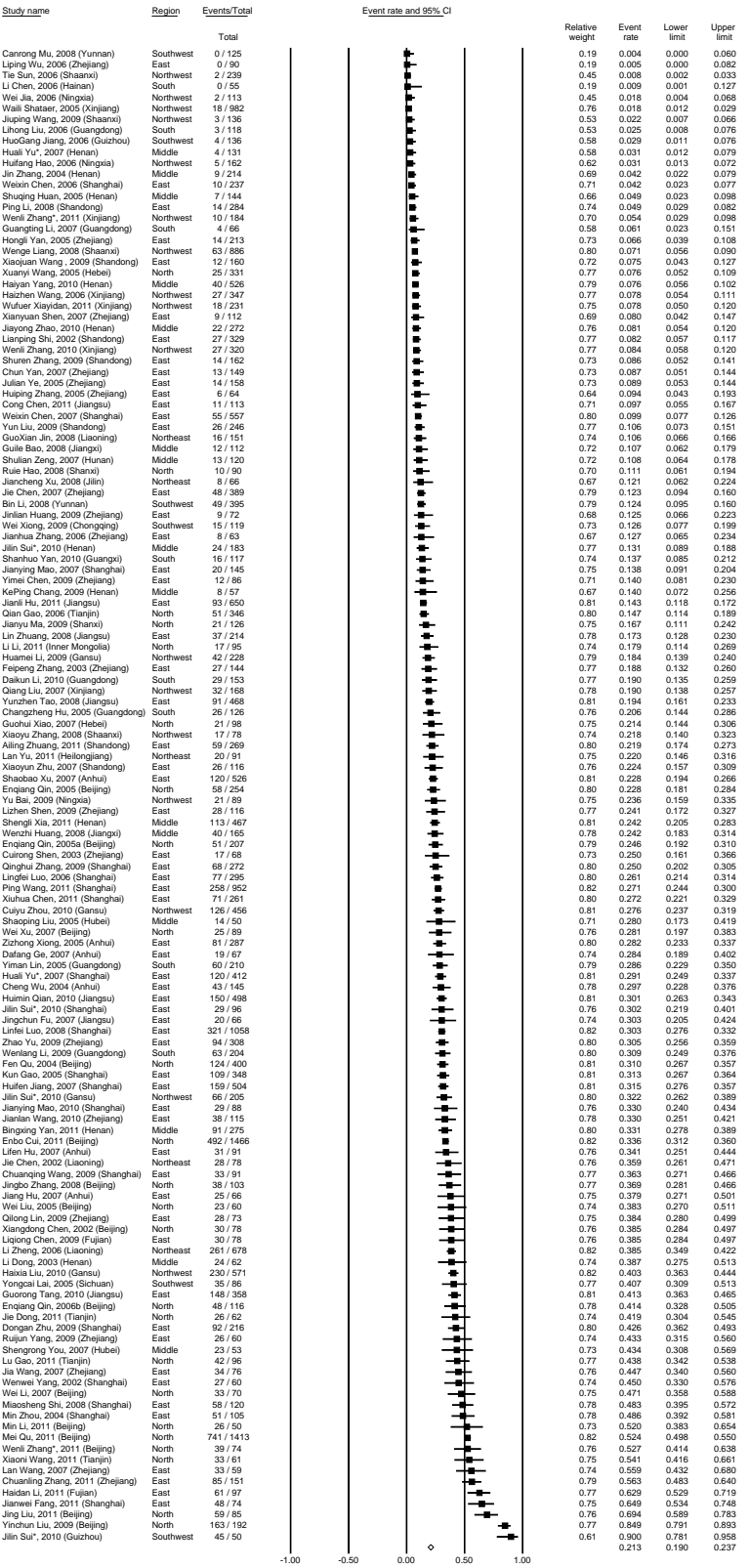

Supplement: Figure S1 — Forest plots of meta-analyses on the prevalence of S. flexneri (A) and S. sonnei (B). (PDF) [file pone.0052515.s001.pdf]
