# Supplementary material for: Causative Species and Serotypes of Shigellosis in Mainland China: Systematic Review and Meta-Analysis
Source: PLoS One. 2012 Dec 20;7(12):e52515. doi: 10.1371/journal.pone.0052515 (PMC3527545; doi:10.1371/journal.pone.0052515)
Supplement: Table S1 — Included and excluded articles in full-text evaluation. (DOC) [file pone.0052515.s002.doc]

**Table S1.** Included and excluded articles in full-text evaluation.

**Included articles (131 in total):**

1. Chen J, Liu H, Luan Y (2002) Serotype distribution and drug resistance analyses of Shigella isolated in Shenyang during past 3 years (in Chinese). Occupation and Health 18: 39-41.

2. Chen X, Lin L, Zhang Y (2002) Pathogen detection and drug resistance of accurate shigellosis in summer of 2001 (in Chinese). Chinese Journal of Medical Laboratory Technology 3: 205-207.

3. Yang W, Ying C (2002) Drug resistance analyses of 60 Shigella isolates (in Chinese). Shanghai Journal of Medical Laboratory Sciences 17: 241-242.

4. Dong L (2003) Analysis on Antibiotic Resistance of 62 Strains Shigella (in Chinese). Journal of Practical Medical Techniques 10: 459-460.

5. Shi L, Lu X, Wang C (2003) Epidemiological investigation of bacillary dysentery in construction workers in Weifang (in Chinese). Chinese Journal of Sanitary Supervision and Health 1: 91-92.

6. Tan C, Cen L, Gu H (2003) The bacterial spectrum and drug resistance of bacillary dysentery (in Chinese). Zhejiang Clinical Medical Journal 5: 661-662.

7. Zhang F (2003) Epidemiological typing and drug resistance analyses of 144 Shigella isolates (in Chinese). Journal of North China Coal Medical College 5: 283-284.

8. Qu F, Bao C, He J (2004) Distribution and antimicrobial resistance of Shigella spp isolated from diarrheal patients between 1993 and 2002 in Beijing (in Chinese). Chinese Journal of Antibiotics 29: 671-674.

9. Wu C (2004) Analysis of the bacterium genus distribution and drug resistance of dysentery bacilli in children (in Chinese). Journal of Anhui Health Vocational & Technical College 3: 64-66.

10. Zhang J, Xia S, Ma H, Wang J (2004) A study on features of dysentery and its pathogens in Henan Province (in Chinese). Chinese Journal of Health Laboratory Technology 14: 45-47.

11. Zhou M, Huang Y, Sun J (2004) Serotyping and susceptibility test of 105 Shigella spp strains (in Chinese). Shanghai Journal of Medical Laboratory Sciences 19: 301-303.

12. Gao K (2005) Serotyping and antibiotics resistance of Shigella spp strains (in Chinese). Journal of Clinical Transfusion and Laboratory Medicine 7: 85-87.

13. Hu C, Huang X (2005) Serological and drug resistance analyses of 126 shigellosis cases in Jiangmen, Guangdong province (in Chinese). New Chinese Medicine 36: 24-25.

14. Huan S, Xu Q, Liu C (2005) Characteristics of shigellosis cases and related pathogens in rural of Sui county (in Chinese). Henan Journal of Preventive Medicine 16: 27-28.

15. Lai L (2005) Drug resistance analyses of 86 Shigella isolates treated by 17 antibiotics (in Chinese). Journal of Practical Medical Techniques 12: 2718-2719.

16. Lin Y, Qiu Y, Zhang Q (2005) Distribution of enteric pathogens in employees of public food service in Shenzhen (in Chinese). China Tropical Medicine 5: 124-125.

17. Liu H, An F (2005) Serogroup distribution and drug resistance characteristics of Shigella isolated in Wuwei area, Gansu province during 2005-2007 (in Chinese). Journal of Clinical Internal Medicine 26: 139-140.

18. Liu S, Liu X, Quan B, Zhang H (2005) Etiology of infectious diarrhea in children (in Chinese). Chinese Journal of Microecology 17: 36-37.

19. Liu W, Sun P (2005) Serotyping and analysis of drug sensitive testing for 60 strains of dysentery bacillus of Haidian district Beijing in 2004 (in Chinese). Foreign Medical Sciences 26: 25-27.

20. Qin E, Zhao M, Zhou Z (2005) Analysis of the group and the rate of drug sensitivity in 254 strains of dysenteric bacilli (in Chinese). Chinese Journal of Practical Internal Medicine 25: 617-618.

21. Qin E, Zhou Z, Zhao M, Wang N (2005) Analysis of the distribution and the rate of drug sensitivity with acute bacterial dysentery in Beijing in 2004 (in Chinese). Journal of Clinical Internal Medicine 22: 189-190.

22. Waili S, Xia Y, Mu T, A D, Ku L, et al. (2005) Serotype distribution and drug resistance of 982 Shigella isolates in Xinjiang province during 2002-2003 (in Chinese). Endemic Disease Bulletin 20: 78.

23. Wang X, Du L, Von Seidlein L, Xu Z, Zhang Y, et al. (2005) Occurrence of shigellosis in the young and elderly in rural China: results of a 12-month population-based surveillance study. Am J Trop Med Hyg 73: 416-422.

24. Xiong Z, Ye L, Xu Y (2005) Variance of serogroups in Shigella and clinical features of patients infected by those strains (in Chinese). Chinese Journal of Clinical 8: 398-399.

25. Yan H, Lv Y (2005) Serogroup and drug resistance analyses of 213 cases of pediatric Shigella infections (in Chinese). Journal of Laboratory Medicine and Clinical 2: 210-211.

26. Ye J, Zhu S, Mo S (2005) Analysis on Distribution of Shigella Flora and Drug Resistance in 2003 in Partial Regions of Zhejiang Province (in Chinese). Disease Surveillance 20: 80-81.

27. Zhang H, Chen Q (2005) Pathogenic detection of Shigella from diarrhea children (in Chinese). China Tropical Medicine 5: 1537-1587.

28. Chen L, CHen H, Mo C (2006) The distribution of 55 strains of Shigella and results of drug sensitivity tests (in Chinese). China Tropical Medicine 6: 2049.

29. Chen W, Wu J, Yu W (2006) Study on the distribution and the level of resistance to drugs' of Shigella in Jiading district in 2004 (in Chinese). Modern Preventive Medicine 33: 1663-1664.

30. Gao Q (2006) Analysis on the constitution of pathogenic bacteria and their susceptibility in children patients with diarrhea in Tianjin aera (in Chinese). Acta Academiae Medicinae CPAPF 15: 244-246.

31. Hao H, Chen Y, Zhao S (2006) Etiological and drug resistance analyses of pediatric enteric infections (in Chinese). Ningxia Medical Journal 28: 781-783.

32. Jia W, Zhou X, Xiao Y (2006) Serogroup distribution and drug resistance analyses of Shigella isolates (in Chinese). Ningxia Medical Journal 28: 554-556.

33. Jiang H, Xiao Y (2006) Serological typing and drug resistance analyses of 136 Shigella isolates (in Chinese). Acta Academiae Medicinae Zunyi 29: 375-377.

34. Liu L, Wang H, Li X (2006) Epidemiological Investigation on 118 Strains of Shigella and Drug Sensitivity Analysis in Clinic (in Chinese). Chinese Primary Health Care 20: 66-67.

35. Luo L, Wang X, Liu J, Wang P (2006) Seasonal Distribution of Shigella and Serum Typing in Minghang District of shanghai in 2004 (in Chinese). Occupation and Health 22: 1848-1849.

36. Qing E, Zhang X (2006) Analysis of the group distribution and drug sensitivity in 116 patients with shigellosis (in Chinese). Beijing Medical Journal 28: 609-610.

37. Sun T, Xue C (2006) Serotype distribution and drug resistance analyses of 506 clinical Shigella isolates (in Chinese). Chinese Journal of Coal Industry Medicine 9: 230.

38. Wang H, Niu X, Yi Y, Zhao L (2006) Enteric pathogen detection in summer of Korla during 2002-2005 (in Chinese). Endemic Diseases Bulletin 21: 77-78.

39. Wu L (2006) Serotype distribution and drug resistance detection of Shigella isolates in Xianju county, Zhejiang province (in Chinese). Disease Surveillance 21: 691.

40. Zhang J, Wang G, Hu L (2006) Epidemiological and drug resistance analyses of Shigella infections in urban of Shaoxing (in Chinese). Strait Journal of Preventive Medicine 12: 21-22.

41. Zheng L (2006) Drug resistance of 1004 diarrhea pathogens isolated in Dadong area, Shenyang during 2001-2003 (in Chinese). Practical Pharmacy and Clinical Remedies 9: 48-49.

42. Chen J, Zhu J, Jin Q (2007) Bacterial spectrum and drug resistance of bacillary dysentery in Hangzhou area 1996-2005 (in Chinese). Chinese Journal of Nosocomiology 17: 1452-1454.

43. Chen W (2007) Serogroup and serotype distribution of Shigella strains isolated in Jiading district, Shanghai (in Chinese). Journal of Modern Laboratory Medicine 22: 48-50.

44. Fu J, JIe Y, XIao Y (2007) Serological study of Shigella strains isolated in Wuxi (in Chinese). Modern Preventive Medicine 34: 1357-1360.

45. Ge D, Chen D, Jiang L, CHen J (2007) Surveillance on bacterial dysentery in the City of Maanshan and analysis on its drug resistance (in Chinese). Disease Surveillance 22: 96-97.

46. Hu J, Chen D, Xia F (2007) Etiological and drug resistance analyses of 171 bacterial diarrhea cases (in Chinese). Journal of Bengbu Medical College 32: 616-617.

47. Hu L, Li X (2007) Detection of Shigella producing beta-lactamases and analysis of their gene types (in Chinese). Acta Universitatis Medicinalis Anhui 42: 87-90.

48. Jiang H, Mao J, Jin K (2007) Epidemiological study of shigellosis in Baoshan district, Shanghai in 2005 (in Chinese). Shanghai Journal of Preventive Medicine 19: 156-157.

49. Li G, Lin Z, Ke S (2007) Research of hospital infectious diarrhea flora and changes of drug resistance (in Chinese). Jilin Medical Journal 28: 1831-1833.

50. Li W, Li S, Zheng X (2007) Serotyping and susceptibility test of 70 Shigella spp strains (in Chinese). Chinese Journal of Nosocomiology 17: 1455-1457.

51. Liu Q, Dong S (2007) Serotype distribution and drug resistance analyses of shigellosis in Bohu county during 2000-2006 (in Chinese). Occupation and Health 23: 2194-2195.

52. Mao J, Jiang H, Yuan G (2007) Epidemiological analyses of shigellosis in Baoshan district, Shanghai in 2004 (in Chinese). Shanghai Journal of Preventive Medicine 19: 106-107.

53. Shi X, Xiao C, DIng Z (2007) Analysis of the distribution and the rate of drug sensitivity of 112 strains of Shigella bacteria (in Chinese). Chinese Journal of Microecology 19: 546-547.

54. Wang J, Wu M, Hou Q, Chen S (2007) Clinical characteristics and drug resistance of pediatric shigellosis (in Chinese). Zhejiang Medical Journal 29: 818-820.

55. Wang L, Xu J, Li H (2007) Serogroup distribution and drug resistance of pediatric shigellosis in Xiaoshan hospital in 2006 (in Chinese). Chinese Primary Health Care 21: 87-88.

56. Xiao G, Ge F (2007) Bacterial species distribution and drug sensitivity in children acute bacillary dysentery (in Chinese). Chinese Journal of Primary Medicine and Pharmacy 14: 1612-1613.

57. Xu S, Wu X, Shen Q (2007) The situation of pathogenic bacteria and drug sensitivity of acute diarrhea (in Chinese). Anhui Medical and Pharmaceutical Journal 11: 719-720.

58. Xu W, Wu W, Li S, Han Q, SHao X (2007) Epidemiological analysis for shigellosis during 2003 to 2006 in Chaoyang District, Beijing (in Chinese). Capital Journal of Public Health 1: 249-251.

59. Yan C, Liu C, Li X, Ge X (2007) Drug resistance analyses of Shigella isolated from pediatric infectious diarrhea patients (in Chinese). Journal of Wenzhou Medical College 37: 407-408.

60. You S (2007) Infection, drug resistance and genotype of Shigella spp isolated from patients with community acquired diarrhea (in Chinese). Acta Medicinae Universitatis Scientiae et Technologiae Huazhong 36: 658-662.

61. Yu H, CHang Z, Zhang L, Zhang J, Li Z, et al. (2007) Analysis on the status of Shigella spp antimicrobial resistance through data from the National Shigellosis Surveillance System in China, in 2005 (in Chinese). Chinese Journal of Epidemiology 28: 370-373.

62. Zen S (2007) The distribution and drug resistance of Shigella in 120 patients in Qidong County of Hunan Province (in Chinese). China Tropical Medicine 7: 266.

63. Zhu X (2007) Etiological study of infectious diarrhea in Linyi (in Chinese). Occupation and Health 23: 1321-1323.

64. Bao G (2008) Pathogen distribution and drug resistance analyses of infectious diarrhea (in Chinese). Experimental and Laboratory Medicine 26: 523-524.

65. Hao R, Zhang Q, Zhao R (2008) Serotype distribution and drug resistance analyses of Shigella in Shanxi province (in Chinese). Shanxi Medical Journal 37: 251.

66. Huang W, Li S (2008) Analysis of serotype and drug resistance of pathogenic bacterium in bacillary dysentery (in Chinese). International Journal of Laboratory Medicine 29: 873-874.

67. Jin G, Wang L, Liu J, Fan J, Wang N, et al. (2008) Pathogen detection and drug resistance analyses from accurate diarrhea patients (in Chinese). Chinese Journal of Public Health 24: 370.

68. Li B, Shen J, Wu X, Xiao S, Ni L (2008) Serogroup distribution and drug resistance analyses of pediatric enteric bacterial infections (in Chinese). Chinese Journal of Primary Medicine and Pharmacy 15: 2034-2035.

69. Li P, XU S, Liu Y, Wang Y, Jin Y, et al. (2008) Serotype distribution and drug resistance analyses of 284 Shigella isolates (in Chinese). Shandong Medical Journal 48: 97-98.

70. Liang W, Zhang K, Jia M (2008) Analysis of distribution and change of Shigella bacteria in Baoji from 1997 to 2006 (in Chinese). Practical Preventive Medicine 15: 1577-1579.

71. Luo L, Wang X, Liu J (2008) Analysis of the distribution and vicissitude of Shigella in Minhang district of Shanghai from 2003 to 2006 (in Chinese). Modern Preventive Medicine 35: 1564-1565.

72. Mu C (2008) Etiological and drug resistance analyses of accurate pediatric diarrhea (in Chinese). Chinese Journal of Medicinal Guide 10: 1233.

73. Shi M, Chen W, Shen J (2008) Serotype distribution and drug resistance analyses of Shigella isolates (in Chinese). Journal of Clinical and Experimental Medicine 7: 94-95.

74. Tao Y, Zhu L, Ding Y (2008) The resistance monitoring inspection on Shigella with which children infected in Suzhou (in Chinese). Chinese Journal of Microecology 20: 60-61.

75. Xu J, Zhou Q, Zheng Y (2008) Surveillance of bacterial resistance and distribution of 101 bacterial pathogens isolated from children with diarrhea (in Chinese). Maternal and Child Health Care of China 23: 863-864.

76. Zhang J, Cui J, Wang L (2008) Change in strain types and drug-susceptibility of Shigella in Xicheng district of Beijing from 2005 to 2007 (in Chinese). Chinese Journal of Zoonoses 24: 982-984.

77. Zhang X, Wu S, Li Y, Wang C (2008) Surveillance and analysis of bacterial pathogens of infectious diarrhea in the city of Xi’an from 2003 to 2007 (in Chinese). Disease Surveillance 23: 34-36.

78. Zhuang L, Qian H, Tan Z (2008) Epidemiological study of shigellosis in Jiangsu province in 2007 (in Chinese). Modern Medical Journal 36: 439-442.

79. Bai Y, Hou S (2009) Serotype distribution and drug resistance analyses of 89 Shigella isolates from diarrhea patients (in Chinese). Journal of Ningxia Medical University 31: 104-105.

80. Chang K, Huang D, HUANG D-yTSH, Xinxiang, Henan, et al. (2009) A dynamic analysis on the pathogenic bacteria and drug sensitivity of the bacterial dysentery among children and its implications (in Chinese). China Clinical Practical Medicine 3: 31-33.

81. Chen L (2009) Etiological and drug resistance analyses of 198 bacterial diarrhea cases (in Chinese). China Foreign Medical Treatment 10: 159.

82. Chen Y (2009) Analysis of distribution and drug resistance of Shigella spp about children's intestinal isolates in Lianxi urburn area (in Chinese). Chinese Journal of Health Laboratory Technology 19: 1859-1860.

83. Huang J (2009) Serotype distribution and drug resistance analyses of 72 Shigella isolates (in Chinese). The Journal of Practical Medicine 25: 472-473.

84. Li W, Che A (2009) 1091 Cases of Bacterial Diarrhea Pathogen Distribution and Antibiotic Resistance Analysis (in Chinese). Chinese Journal of Medicinal Guide 11: 478-479.

85. Lin Q, Xia B, Chen B (2009) Analysis of pathogenic bacteria and drug resistance of bacterial diarrhea in Zhoushan islands′ children (in Chinese). Chinese Journal of Health Laboratory Technology 19: 1853-1854.

86. Liu Y, Yi M, Li J, Jia H (2009) Analysis on the serotypes and antimicrobial-susceptibility of Shigella and salmonella spp isolates (in Chinese). Journal of China-Japan Friendship Hospital 23: 213-215.

87. Liu Y, Zhang M, Chen S, ZHANG M, CHEN S (2009) Analysis of the Bacterial Types and the Drug Resistance of the Shigella in Jinan During the Years from 2003 to 2007 (in Chinese). Journal of Modern Laboratory Medicine 24: 109-111.

88. Ma J, Zhang Q, Hao R, Liang Z, Zhao R, et al. (2009) Serogroup distribution and drug resistance analyses of 126 Shigella isolates (in Chinese). Journal of Shanxi Medical College for Continuing Education 19: 78-79.

89. Shen L, Xu F, Yu L (2009) Serotyping and drug resistance of Shigella circulated in Wenzhou, Zhejiang province (in Chinese). Disease Surveillance 24: 334-336.

90. Wang C, He L, Wang A, Zhang W (2009) Characteristics of bacterial pathogens associated with community-acquired pediatric diarrhea in Shanghai in 2007 (in Chinese). Journal of Microbes and Infection 4: 97-99.

91. Wang J, Wang D, Nie Q, Wang P, Huang Z (2009) Bacterial culture on stools in 496 patients with diarrhea and antimicrobial resistance (in Chinese). Chinese Journal of Infection Control 8: 413-416.

92. Wang X, Yi Y, Chen Y (2009) Drug resistance analyses of 160 Shigella isolates (in Chinese). Chinese Journal of Experimental and Clinical Infectious Diseases (Electronic Version) 3: 436-438.

93. Xiong W, Li N, Zheng P (2009) Clinical study on the bacterial distribution, drug sensitivity and clinical significance in children with acute bacterial dysentery (in Chinese). Chongqing Medicine 38: 949-950.

94. Yang R, Xu B, Cao G (2009) Distribution of serotyping of Shigella in Quzhou from 2007 to 2008 (in Chinese). Chinese Journal of Health Laboratory Technology 19: 2386-2387.

95. Yu Z, Luo Y, Qin S (2009) Analysis on epidemiological characteristics of bacillary dysentery in Zhejiang province,2004-2007 (in Chinese). China Preventive Medicine 10: 356-358.

96. Zhang Q, Chun Y, Zhu P, Huang Z (2009) Epidemiological characteristics of shigellosis cases in Songjiang district, Shanghai during 2004-2007 (in Chinese). Shanghai Journal of Preventive Medicine 21: 66-67.

97. Zhang S, Ren G, Wang W (2009) Serotype distribution and drug resistance of Shigella isolates from hospital acquired shigellosis (in Chinese). Shanxi Medical Journal 38: 235-237.

98. Zhu A, Sun J, Fan H, SUN J-y, FAN H-q (2009) Antimicrobial resistance and extended-spectrum β-lactamases genotypes of Shigella isolates in Shanghai (in Chinese). Chinese Journal of Infection and Chemotherapy 9: 126-128.

99. Li D, Huang Y, Fu W (2010) Pathogenic bacteria of infantile diarrhea and their drug resistance (in Chinese). International Journal of Laboratory Medicine 31: 322-324.

100. Liu H, Liu J, Liu X (2010) Analysis on bacillary dysentery surveillance in Gansu province from 2005 to 2009 (in Chinese). Chinese Journal of Health Laboratory Technology 20: 2553-2554.

101. Mao J, Yuan G, Cao G (2010) Analysis on Detection Results of Bacillary Dysentery in National Surveillance Site in Baoshan District of Shanghai in 2009 (in Chinese). Occupation and Health 26: 1866-1868.

102. Qian H, Zhuang L, Dong C (2010) Analysis on the serotype and antimicrobial resistance of Shigella spp during 2008~2009 in Jiangsu Province (in Chinese). Jiangsu Journal of Preventive Medicine 21: 15-17.

103. Sui J, Zhang J, Sun L, Chang Z (2010) Surveillance of bacillary dysentery in China,2009 (in Chinese). Disease Surveillance 25: 947-950.

104. Tang G, Mei B (2010) Serogroup distribution and drug resistance analyses of pediatric shigellosis (in Chinese). Jiangsu Medical Journal 35: 2577-2578.

105. Wang J, Zhu H (2010) Analysis of testing results of 178 Shigella and Salmonella in Haining (in Chinese). Chinese Journal of Health Laboratory Technology 20: 2933-2934.

106. Yan S, Zhuo Y (2010) Etiological and drug resistances analyses of pediatric bacterial infectious diarrhea in Qinzhou area (in Chinese). Laboratory Medicine and Clinic 7: 848-850.

107. Yang H, Duan GC, Zhang W, Zhu J, Xi Y, et al. (2010) Serotypes characterization and antimicrobial resistance on Shigella isolated from the Sui county during 2001-2008 (in Chinese). Chinese Journal of Epidemiology 31: 351-353.

108. Zhang W, Li X, Yang H (2010) Strain types and resistance of Shigella strains isolated in children of Urumqi (in Chinese). Chinese Journal of Public Health 26: 897-898.

109. Zhao J, Luo Q, Li M, Huang L (2010) Etiology of shigellosis in Henan province,2008 (in Chinese). Disease Surveillance 25: 955-957.

110. Zhou C, Wang K (2010) Surveillance of shigellosis in Chengguan district, Lanzhou, Gansu province during 2005-2009 (in Chinese). Health Vocational Education 28: 117-118.

111. Chen C, Xu H, Yao X (2011) Epidemiological and etiological characteristics of bacillary dysentery in Changzhou city from 2004 to 2009 (in Chinese). Acta Universitatis Medicinalis Nanjing(Natural Science) 31: 274-278.

112. Chen X, Zhang Y, Xu H (2011) Serotype Distribution and Drug Resistance of 261 Shigella Strains (in Chinese). Occupation and Health 27: 1845-1846.

113. Cui E, Guo T, Bao C (2011) Distribution and antimicrobial resistant tendency of Shigella species and serotype in diarrhea patients during 18 years (in Chinese). Chinese Journal of Nosocomiology 21: 3526-3528.

114. Dong J, Gao X, Chen J (2011) Distribution of Shigella Enterotoxins Genes and PFGE Molecular Typing of Shigella Strains in Tianjin (in Chinese). Journal of Environment and Health 28: 111-113.

115. Fang J, Wang F (2011) Epidemiological characteristics and antibiotics resistance: analysis of 74 cases of bacillary dysentery (in Chinese). Heilongjiang Medicine and Pharmacy 34: 20-21.

116. Gao L, Dong J (2011) Surveillance of shigellosis in Tianjin during 2007-2009 (in Chinese). Chinese Journal of Preventive Medicine 45: 173-174.

117. Hu J, Qian H, Zhuang L (2011) Analysis on epidemic characteristics of bacillary dysentery in Jiangsu province from 2004 to 2010 (in Chinese). Chinese Preventive Medicine 12: 419-422.

118. Li H, Luo C, Xie Y, Zhang Z (2011) Analysis of Shigella spp surveillance in Fujian province from 2005 to 2009 (in Chinese). Chinese Journal of Health Laboratory Technology 21: 694-695.

119. Li L (2011) Serogroup distribution and drug resistance analyses of 95 Shigella isolates (in Chinese). Journal of Baotou Medical College 27: 24-25.

120. Li M, Cai W, Bai X, Ren L (2011) Epidemiological analysis for bacillary dysentery in Shijingshan district of Beijing during 2004--2010 (in Chinese). Capital Journal of Public Health 5: 63-67.

121. Liu J, Miao Y, Wang L, Cui J (2011) Analysis on Change of Serum Types and Drug Resistance of Shigella in Xicheng District of Beijing from 2008 to 2010 (in Chinese). Occupation and Health 27: 1377-1379.

122. Qu M, Liu G, Zhang X (2011) Distribution of different serotypes and analysis of virulence genes in Shigella species in Beijing from 2004 to 2010 (in Chinese). Chinese Journal of Health Laboratory Technology 21: 1850-1853.

123. Wang P, Wang X, Luo L (2011) Serotyping of 952 Shigella strains and its resistance analysis in Minhang district of Shanghai city (in Chinese). Occupation and Health 27: 540-542.

124. Wang X, Wei D (2011) Antimicrobial resistance in Shigella spp and based on ERIC typing character crossing two decades in Tianjin (in Chinese). Clinical Focus 26: 775-778.

125. Xia S, Xu B, Huang L, Zhao JY, Ran L, et al. (2011) Prevalence and characterization of human Shigella infections in Henan Province, China, in 2006. J Clin Microbiol 49: 232-242.

126. Xiayidan W, Liu Q, Wai L (2011) Etiological and drug resistance analyses of shigellosis in Xinjiang province in 2006 (in Chinese). Bulletin of Disease Control and Prevention 26: 66-67.

127. Yan B, Ma W, Sun Y, Li J (2011) Antimicrobial resistance in Shigella isolates from children under six years of age with acute diarrhea in Puyang city, 2007 to 2009 (in Chinese). Chinese Journal of Health Laboratory Technology 21: 480-483.

128. Yu L, Zhao Y, Huang H (2011) Etiological surveillance of shigellosis in Heilongjiang province during 2005-2010 (in Chinese). Chinese Journal of Public Health Management 27: 390-392.

129. Zhang C, Zhang G, Wang H (2011) Analysis of the distribution and the resistance of 151 strains of Shigella group (in Chinese). Chinese Journal of Antibiotics 36: 311-314.

130. Zhang W, Luo Y, Li J, Lin L, Ma Y, et al. (2011) Wide dissemination of multidrug-resistant Shigella isolates in China. J Antimicrob Chemother 66: 2527-2535.

131. Zhuang A, Liu S, Guan E (2011) Pathogen detection from infectious diarrhea patients in clinics (in Chinese). Qingdao Medical Journal 43: 35-36.

**Excluded articles (172 in total):**

1. **Repeated data**

1. Peng X (2004) Investigation of the flora distribution and antimicrobial susceptibility of Shigella in the patients with acute bacterial diarrhoea (in Chinese). Journal of China-Japan Friendship Hospital 18: 332-334.

2. Qu F, Bao C, Cui E (2004) Features of enteric pathogenic bacteria in Beijing in recent 4 years (in Chinese). Infections Disease Information 17: 26-28.

3. Qu F, Mao Y, Bao C (2005) Antimicrobial resistance of bacterial pathogens associated with diarrheal patients in Beijing during 2000—2003 (in Chinese). Chinese Journal of Laboratory Medicine 28: 384-386.

4. Chen W (2006) Emergence of three new sub-serotypes of Shigella and their vicissitude in 11 years in Jiading District (in Chinese). Chinese Journal of Health Laboratory Technology 16: 1167-1169.

5. Hao H, Chen Y, Zhao S (2006) Etiological and drug resistance analyses of pediatric infections (in Chinese). Journal of Ningxia Medical College 28: 421-423.

6. von Seidlein L, Kim DR, Ali M, Lee H, Wang X, et al. (2006) A multicentre study of Shigella diarrhoea in six Asian countries: disease burden, clinical manifestations, and microbiology. PLoS Med 3: e353.

7. Yu W (2006) Serological analyses of Shigella isolated in Jiading district, Shanghai during past five years (in Chinese). Modern Preventive Medicine 33: 1971-1973.

8. Hu L, Chen J, Li J (2007) Distribution and Resistance to Antibiotics of Shigella in Anhui Province (in Chinese). Chinese Journal of Nosocomiology 17: 218-220.

9. Liu L, Qu F, Cui E (2007) Distribution and resistance of enteric pathogenic bacteria in Beijing area from 2005 to 2006 (in Chinese). Chinese Journal of Antibiotics 32: 745-747.

10. Hu S, Junan, China (2008) Investigation of diarrhea pathogens in Junan County (in Chinese). Chinese Journal of Health Laboratory Technology 18: 333-334.

11. Li F, Wang C, Zhang X (2008) Etiological surveillance of infectious diarrhea in Xi'an during 2003-2007 (in Chinese). Practical Preventive Medicine 15: 1590-1591.

12. Li H, Luo C, Xie Y (2009) Etiological surveillance of bacillary dysentery in Fujian province during 2005-2008 (in Chinese). Strait Journal of Preventive Medicine 15: 30-31.

13. Liu Y, Zhang B, Wang J (2009) Analysis of types and antibiotic susceptibility of 246 Shigella isolates (in Chinese). Chinese Journal of Health Laboratory Technology 19: 1113-1115.

14. Zhang M (2009) Serological and drug resistance analyses of Shigella (in Chinese). Public Medical Forum Magazine 13: 1022-1023.

15. Luo C, Xie Y, Li H (2010) Surveillance and Analysis of Bacillary Dysentery from 2005 to 2009 in Fujian,China (in Chinese). Strait Journal of Preventive Medicine 16: 17-19.

16. Zhang J, Li D, Cui J (2010) Detection and analysis of virulence-associated genes in Shigella species in Xicheng district of Beijing from 2005 to 2007 (in Chinese). Chinese Journal of Zoonoses 26: 269-271.

17. Li H, Luo C, Xie Y (2011) Drug resistance analyses of Shigella isolates in Fujian province during 2005-2009 (in Chinese). Strait Journal of Preventive Medicine 17: 87-88.

1. **Reports of outbreak**

1. Zhang G, Yu D, Wang P (2010) Epidemic Shigella in Gansu province, 2005-2008 (in Chinese). Chinese Journal of Natural Medicine 12: 271-273.

1. **Study period out of 2001-2010**

1. Gao J (2001) Surveillance report of bacterial pathogens causing diarrhea in Hohhot railway area (in Chinese). Nei Moivgol Medical Journal 33: 67-68.

2. Gao X, Wang Y, Ruan Z (2001) Etiological study of 1200 cases of infectious diarrhea in Huimin county (in Chinese). Occupation and Health 17: 101-102.

3. Luo X, Feng X (2001) Etiological study of 3253 stool specimen of diarrhea patients in intestinal clinic (in Chinese). Guangdong Medical Journal 22: 697-698.

4. Shi W, Qiu X (2001) Analysis of drug resistances of Shigella isolated in Shanghai railway area (in Chinese). Railway Medical Journal 29: 328-329.

5. Yang J, Zen L, Chen X (2001) Serological and drug resistance analyses of 51 Shigella isolates (in Chinese). Journal of Youjiang Medical College For Nationalities 11: 278.

6. Zhou W, Wei Y, Feng X (2001) Bacteria detection in 1656 pediatric diarrhea cases (in Chinese). Henan Journal of Preventive Medicine 12: 20-21.

7. Gan H (2002) Analysis of drug resistances of Shigella isolated in Shanghai Railway Hospital during 1999-2001 (in Chinese). Herald of Medicine 21: 447.

8. Guo W, Gou X, Chen Q (2002) Study of clinical and etiological characteristics of shigellosis in surveillance sites (in Chinese). Henan Journal of Preventive Medicine 13: 81-83.

9. Huang W (2002) Etiological study of diarrhea cases in Xining area (in Chinese). Chinese Journal of Nosoconmiology 12: 80-83.

10. Qu F, Wang H, Cui E (2002) Distribution and resistance of enteric pathogenic bacteria in Beijing area (in Chinese). Chinese Journal of Infectious Diseases 20: 25-27.

11. Yang M, Zhang J, Chang R (2002) Etiological study of 2489 infectious diarrhea cases (in Chinese). Shaanxi Medical Journal 31: 197-198.

12. Yang W (2002) Etiological and drug resistance analyses of 688 infectious diarrhea cases (in Chinese). Shaanxi Medical Journal 31: 809-810.

13. Zhao G, Ju C (2002) Serum distribution and drug sensibility trial of 85 dysentery bacteria strains (in Chinese). Henan Medical Research 11: 343-344.

14. Huang Q, Wang R (2003) Serological and drug resistance analyses of 115 Shigella isolates in Huaibei (in Chinese). Chinese Journal Of Medical Laboratory Technology 4: 64-65.

15. Cai Y, Zhang Z, Cai Z (2004) Clinical analysis on Pediatric bacterial dysentery in Nanchang district in 2002 (in Chinese). Pediatric Emergency Medicine 11: 93-95.

16. Wu J (2004) Analysis of results on Salmonella and Shigella in Jiading district (in Chinese). Modern Preventive Medicine 31: 131-132.

17. Zhao M (2004) Investigation on serotypes and antibiotic resistance of Shigella in Pinggu district (in Chinese). Beijing Medical Journal 26: 183-185.

18. Zhu J, Duan G, Zhang M (2004) Study on the antimicrobial resistance in Shigella spp (in Chinese). Henan Journal of Preventive Medicine 15: 5-8.

19. Ding J, Guo Z, Chen L (2005) Six-year bacterial species distribution and drug sensitivity in childhood acute bacillary dysentery: An investigation of 290 cases (in Chinese). The Chinese Journal of Contemporary Pediatrics 7: 54-56.

20. Miu X (2005) Systematic analysis of shigellosis surveillance in Wuxi during past 25 years (in Chinese). Occupation and Health 21: 125-126.

21. Ren D, Zhang Y, Zhou R (2005) Epidemiological Analysis on Bacillary Dysentery in Jiaozuo City from 1985 to 2004 (in Chinese). Literature and Information On Preventive 11: 597-598.

22. Shao C, Zhang D (2005) Serological, drug resistance and plasmids analyses of Shigella (in Chinese). Occupation and Health 21: 529-530.

23. Shi Y, Wang H, Kang L (2005) Change of bacteria and drug resistance pattern causing bacillary dysentery (in Chinese). Journal of Chinese Modern Medicine 2: 751-753.

24. Tang W (2005) Bacterial Clump Distribution and Drug Resistance Rate of 154 Children With Bacterial Dysentery (in Chinese). Practical Preventive Medicine 12: 1313-1314.

25. Wang C, Xie G, Zhao Q (2005) Serological and drug resistance analyses of pediatric shigellosis (in Chinese). Foreign Medical Sciences 26: 252-254.

26. Wang X, Wang P, Liu J (2005) Epidemiological Analysis on 194 Strains of Shigella and Drug Sensitivity Investigation (in Chinese). Disease Surveillance 20: 71-72.

27. Xu J, Liu W, Ku W (2005) Pathogen Distribution and Drug Resistance in Diarrhea (in Chinese). Chinese Journal Medical Laboratory Technology 6: 325.

28. Chen X (2006) The analysis of the distribution and the susceptivity test of 183 strains of Shigella genes (in Chinese). Chinese Journal of Microecology 18: 315-317.

29. Hu X, Jiao D (2006) Serological and drug resistance analyses of Shigella isolated in Dazhi area during past decade (in Chinese). Chinese magazine of clinical medicinal professional research 12: 678.

30. Jia D (2006) Serological and drug resistance analyses of rural pediatric shigellosis (in Chinese). Chongqing Medicine 35: 549.

31. Li P, Hu B (2006) Disease-burden, serological and drug resistance analyses of shigellosis in Manas county during 1990-2003 (in Chinese). Endemic Diseases Bulletin 21: 33-35.

32. Xia X, Liu B (2006) Analysis on Distribution and Drug-resistance Spectrum Change of Z-H Bacilli (in Chinese). Heilongjiang Medical Journal 30: 42-43.

33. Xu M (2006) Epidemiological analysis of shigellosis in Shaoxing during 1991-2001 (in Chinese). Modern Preventive Medicine 33: 2449.

34. He M, Zhong X (2007) Serological and drug resistance analyses of Salmonella and Shigella isolates in Changzhou (in Chinese). Occupation and Health 23: 2180-2181.

35. Li H, Huang J, Long J, SUN Y-SUG (2007) Analyses of Serotypes and Resistance of Shigella spp (in Chinese). International Medicine & Health Guidance News 13: 67-70.

36. Pu X, Pan J, Meng D, PAN J-c, MENG D-m, et al. (2007) Study on molecular epidemiology of Shigella in Hangzhou district during 1998 to 2005 (in Chinese). Chinese Journal of Epidemiology 28: 206-207.

37. Tian H (2007) Isolation and identification of Shigella from diarrhea patients (in Chinese). Chinese Journal of Public Health 23: 555.

38. Zhang Y, Cao Y (2007) Analysis of the epidemiological characteristics of bacillary dysentery in Zhaotong from 1953 to 2005 (in Chinese). Disease Surveillance 22: 305-306.

39. Ge H, Qin Y, Wu J (2008) Serological and drug resistance study of 63 isolates of Shigella (in Chinese). Qinghai Medical Journal 38: 64-65.

40. Ge Y (2008) Serological, biochemical and drug resistance analyses Shigella isolated in Tonghua during 1977-2002 (in Chinese). Chinese Journal of Health Laboratory Technology 18: 2168.

41. Zhao L, Zhu C (2008) Analysis of distribution and drug resistance of pathogenic bacteria about children’s diarrhea in Chongqing area from 1997 to 2006 (in Chinese). Chinese Journal of Practical Pediatrics 23: 45-49.

42. Jiao F, Kang H, Zhang X, Hao R, KANG H, et al. (2009) Etiological detection and treatment of bacillary dysentery in 416 Chinese children (in Chinese). Chinese Journal of Woman and Child Health Research 20: 175-177.

43. Liu D, Zhang B (2009) Change trend of drug-sensitivity and bacterial clump of children acute bacillary dysentery (in Chinese). Clinical Medicine 29: 34-35.

44. Chen Q, Che C (2010) Research on distribution and drug resistances of bacterial pathogens causing paediatric diarrhea (in Chinese). Jiangxi Medical Journal 45: 1050-1052.

1. **Studies without species data**

1. Ding M, Zhang H, Xu H (2001) Drug resistance study of 141 bacillary dysentery cases and the clinical applications (in Chinese). Journal of Weifang Medical College 23: 39-40.

2. Wang S, Wei S (2001) Clinical and etiological analyses of accurate bacillary dysentery (in Chinese). Journal of Henan Medical College For Staff and Workers 13: 69.

3. Jiang S, Cui X, Yang J (2002) Etiological and drug resistance analyses of 167 pediatric enteric infections (in Chinese). Chinese Journal of Contemporary Pediatrics 4: 313-314.

4. Li S, Meng S, Liang Z (2002) Drug Resistance and Distribution of 1032 strains of Pathogenic Bacteria leading to Diarrhea (in Chinese). Practical Preventire Medicine 9: 474-476.

5. Xiao S, Lin L, Lin C (2002) Study on Diarrhea Etiology in Rural Area of Eastern Fujian (in Chinese). Modern Preventive Medicine 29: 782-783.

6. Bian C, Xiong P, Cai A (2003) Analysis of present situation on the antibiotic resistance of common pathogenic bacteria in nosocomial infection (in Chinese). Medical Journal of Chinese Civil Administration 15: 210-213.

7. Li Y, Zhang M, Wang Y (2003) Analysis of Pathogenic Bacteria in Children with Diarrhea and Antibiotic Resistance (in Chinese). Journal of Practical Medical Techniques 10: 193-195.

8. Lu Q, Xu Y (2003) Pathogen detection from stool of diarrhea patients and drug resistance study (in Chinese). Shanghai Journal of Medical Laboratory Sciences 18: 369-371.

9. Ren F, Zhao X (2003) Etiological and epidemiological study of accurate infectious diarrhea (in Chinese). Preventive Medicine Tribune 2: 404-405.

10. Song Q, Yu J, Zhao G (2003) Analysis of epidemiological investigation on infectious diarrhea (in Chinese). Jiangsu Preventive Medicine 14: 4-6.

11. Chen H, He J, Wu X (2004) The Distribution and Antimicrobial Susceptibility Analysis of 266 Diarrhea Pathogen (in Chinese). West China Medical Journal 19: 419-420.

12. Huang G, Zhao R, You P (2004) Etiological study of 1068 diarrhea cases (in Chinese). Journal of Ningxia Medical College 26: 284-285.

13. Jiang P, Zhi Z (2005) Etiological and drug resistance analyses of pediatric infectious diseases (in Chinese). The Journal of Practical Medicine 21: 1953-1954.

14. Li L, Sun Z, Zhu X, Zhang J (2005) Analysis of antimicrobial resistance of clinical bacteria isolated from county hospitals and tertiary hospitals (in Chinese). Central China Medical Journal 29: 25-28.

15. Liu X, Tang D, Xiao H (2005) Etiological study of 481 diarrhea cases (in Chinese). Literature and Information On Preventive 11: 469-470.

16. Qin E, Zhou Z (2005) Serological and drug resistance analyses of 196 S. flexneri isolates (in Chinese). Journal Of Chinese Modern Medicine 2: 58-59.

17. Wen Z, Lai X (2005) Analysis on Frequency and Drug-resistance of Bacterial Pathogens in Pediatrics (in Chinese). Hubei Journal of Preventive Medicine 16: 9-11.

18. Yang X, Zhang G, Shen Q (2005) Pathogenic analysis of 458 cases in diarrhea disease of inpatient children (in Chinese). Shaanxi Medical Journal 34: 718-720.

19. Zhao R, Zhen Y, Che Q (2005) Distribution of pathogenic bacteria in children with diarrhea and analyses of antibiotic resistance (in Chinese). Chinese Journal of Microecology 17: 278-279.

20. Zuo X, Meng H, Zhang H (2005) Five year dynamic surveillance of infection indices among employees of catering trade in Jing’an district, Shanghai (in Chinese). Chinese Journal of Health Education 24: 29-31.

21. Lu H (2006) Surveillance of enteric pathogens in employees of public food service in Wujiang in 2005 (in Chinese). Journal Of Chinese Modern Medicine 3: 1076-1077.

22. Ma J, Liu J (2006) Clinical and etiological analyses of 452 pediatric infectious diarrhea cases (in Chinese). Public Medical Forum Magazine 10: 312-313.

23. Niu X, Wang H, Chen W (2006) Laboratory analyses of 374 summer diarrhea cases in Bazhou, Xinjiang during 2003-2004 (in Chinese). Endemic Diseases Bulletin 21: 93.

24. Song Q, Song Y, Liu X (2006) Pediatric bacillary dysentery (in Chinese). China and Foreign Medical Journal 4: 38-39.

25. Sun Z, Li L, Zhu X, Ma Y, Li J, et al. (2006) Analysis on antimicrobial resistance of clinical bacteria isolated from county hospitals and a teaching hospital. J Huazhong Univ Sci Technolog Med Sci 26: 386-388.

26. Wang Q (2006) Epidemiological study of community hospital acquired infections (in Chinese). Journal of Qiqihar Medical College 27: 839-840.

27. You J, Zhao Z (2006) Etiological and clinical analyses of pediatric diarrhea (in Chinese). The Journal of Practical Medicine 22: 1797-1798.

28. Zhou J, Liu W (2006) Analysis of Susceptibility Test and Clinical Medication in Treating of 64 Pediatric Patients with Bacillary Dysentery (in Chinese). Journal of Modern Food and Pharmaceuticals 16: 56-57.

29. Bian B (2007) Etiological study of bacillary dysentery cases in middle schools in Linzhou county (in Chinese). Chinese Journal of Health Laboratory Technology 17: 2082.

30. Gao T, Liu G, Li X (2007) Analysis about epidemic situation of dysentery near upon fourteen years in Beijing (in Chinese). Chinese Journal of Preventive Medicine 41: 54-57.

31. Jin H, Ren Y, Lu S (2007) Typing and analysis of virulence genes of Shigella flexneri in Jiyuan (in Chinese). Chinese Journal of Health Laboratory Technology 17: 508.

32. Jin H, Ren Y, Lu X (2007) Analysis on The Resistance of 91 Strains of Shigella flexneri to Drugs in Jiyuan City (in Chinese). Modern Preventive Medicine 34: 2154-2155.

33. Song X, Ma X, Liu Y (2007) Clinical observations of 86 accurate bacillary dysentery cases treated with Rifaximin (in Chinese). Henan Journal of Preventive Medicine 18: 467-468.

34. Xiong Y (2007) Etiological and clinical analyses of 308 pediatric diarrhea cases (in Chinese). Journal Of Chinese Modern Pediatrics 4: 219-222.

35. Ye Y, Wang X, Wang D (2007) Clinical epidemiology of Shigellosis in children from 1996 to 2005 (in Chinese). Chinese Journal of Infection and Chemotherapy 7: 113-115.

36. Zhu X (2007) Study on the result of 40286 person-time practitioners’ physical examination in Linyi from 2004 to 2006 (in Chinese). Journal of Community Medicine 5: 7-9.

37. Dong J, Li Y, Tian G (2008) Etiological and drug resistance analyses of 335 accurate bacillary dysentery cases (in Chinese). Beijing Medical Journal 30: 701.

38. Huang J, Sun Z, Liu Y (2008) Analysis of drug sensitivity of Shigella flexneri in 66 pediatric patients with bacillary dysentery (in Chinese). Modern Preventive Medicine 35: 193-194.

39. Jiang K, Jiang F, Wang Z (2008) Pathogenic Analysis of 600 Stool Samples from Infants with Diarrhea (in Chinese). Occupation and Health 24: 2569-2570.

40. Wang J (2008) Epidemiological characteristics of accurate bacillary dysentery in Jingmen during 2004-2007 (in Chinese). Journal of Public Health and Preventive Medicine 19: 53-54.

41. Yuan Z, Yan B (2008) Drug resistance analyses of enteric pathogens and the clinical applications (in Chinese). Journal of Medical Forum 29: 50-51.

42. Zheng A, Wang L, Li Y (2008) Carriage of Salmonella and Shigella among Healthy Staff Members in the Public Places of Hualong in Shenzhen (in Chinese). Occupation and Health 24: 534-535.

43. Zhou L, Jiang Z, Wang X (2008) Investigation on Bacterial Foodborne Diseases in Mianyang City from 2003 to 2006 (in Chinese). Modern Preventive Medicine 35: 3938-3939.

44. Zhu Z, Li J (2008) Analysis to drug resistance of Dysenteric bacilli: A report of 51 cases (in Chinese). Journal of Bengbu Medical College 23: 477-478.

45. He C (2009) Epidemiological characteristics of bacillary dysentery in Daishan county during 1998-2007 (in Chinese). Zhejiang Journal of Preventive Medicine 21: 32-33.

46. Long Q, Benli X, Guofen Y (2009) Epidemiological study of bacillary dysentery in the army during 1992-2007 (in Chinese). Journal of Preventive Medicine of Chinese People’s Liberation Army 27: 67.

47. Pu Z, Zhao M, Yu H (2009) Etiological study of infectious diarrhea in adults (in Chinese). Chinese Journal for Clinicians 37: 35-37.

48. Shi C (2009) Drug resistance analyses of 178 Shigella isolates and clinical application (in Chinese). Laboratory Medicine and Clinic 6: 532.

49. Wang X (2009) Clinical symptoms and laboratory analyses of 154 accurate diarrhea cases (in Chinese). Chinese Journal of Misdiagnostics 9: 7685-7686.

50. Wei C, Zhai X, Mao H (2009) Bacteria detection and drug resistance analyses in pediatric clinics (in Chinese). Journal of Huaihai Medicine 27: 31-32.

51. Zhang S (2009) Etiological and drug resistance analyses of 110 isolates from bacillary dysentery (in Chinese). Chinese Journal Of Practical Medicine 36: 90-91.

52. Zhao L, Tang H, Shen H (2009) Foodborne diseases in Minhang district, Shanghai, 2006-2007 (in Chinese). Chinese Journal of Public Health 25: 1383-1384.

53. Zhao M, Wan G, Huang Y (2009) Multiplex PCR detection of four common pediatric enteric pathogens (in Chinese). Guangdong Medical Journal 30: 1069-1071.

54. Chen X, Zhang Y, Xu H (2010) Pathogen detection in anal swabs of diarrhea patients from enteric clinic in Minhang district in 2009 (in Chinese). China Practical Medicine 2: 141-142.

55. Dai Y, Yuan B (2010) Surveillance of food-borne diseases in sentinel hospitals in Jiangsu province (in Chinese). China Practical Medicine 5: 266-267.

56. Dou H, Xie X, Zhang X (2010) 2008 Mohnarin Report: Results of Enterobacteriaceae resistance (in Chinese). Chinese Journal of Antibiotics 35: 556-564.

57. Gao K (2010) Serological and drug resistance analyses of clinical Shigella strains isolated during 2002-2008 (in Chinese). Laboratory Medicine 25: 239.

58. Gao X, Gao J (2010) Etiological and drug resistance analyses of 237 diarrhea cases (in Chinese). Laboratory Medicine and Clinic 7: 1210-1211.

59. Jiang G, Liu J, Zhang C (2010) Characteristics of bacillary dysentery epidemic in Kaiyang county during 2004-2009 (in Chinese). China Health Monthly 29: 29-30.

60. Li X, Tian Z, Li B (2010) Summer Diarrhea: an Analysis of 2197 Patients (in Chinese). Acta Academiae Medicinae Qingdao Universitatis 46: 70-72.

61. Liu Y (2010) Etiological and drug resistance analyses of 152 bacillary dysentery cases (in Chinese). Qingdao Medical Journal 42: 187-188.

62. Song J, Pu Z, Zhao M, PU Z-h, ZHAO M-m, et al. (2010) Etiology and drug sensitivity analysis of 533 cases of adult infectious diarrhea in Yantai (in Chinese). World Journal of Infection 10: 42-44.

63. Song J, Pu Z, Zhao M, PU Z-h, ZHAO M-m, et al. (2010) Etiology and drug sensitivity analysis of 533 cases of adult infectious diarrhea in Yantai (in Chinese). Public health and dinical medicine 6: 219-221.

64. Wang SM, Ma JC, Hao ZY, Zhang ZY, Mason C, et al. (2010) Surveillance of shigellosis by real-time PCR suggests underestimation of shigellosis prevalence by culture-based methods in a population of rural China. J Infect 61: 471-475.

65. Xu L, Pang J, Zou X (2010) Epidemiological analyses of bacillary dysentery in Changshu during 2004-2008 (in Chinese). National Medical Frontiers of China 5: 94.

66. Yan W, Xu Y, Yang X, Zhou Y (2010) A hybrid model for short-term bacillary dysentery prediction in Yichang City, China. Jpn J Infect Dis 63: 264-270.

67. Zhan C (2010) Drug resistance analyses of 155 Shigella isolates (in Chinese). Journal of China Traditional Chinese Medicine Information 2: 89.

68. Zhao Z, Li R (2010) Analysis of Clinical Common Pathogenic Bacteria and Their Drug Resistance (in Chinese). Anti-Infection Pharmacy 7: 121-124.

69. Zheng Y, Zhang P, Liu L (2010) Pathogenic investigation in diarrhea disease of inpatient infants and children (in Chinese). Chongqing Medicine 39: 844-846.

70. Qing L, Zhanwei G, Chunmei Y (2011) Epidemiological study of infectious diseases outbreaks in the army during 2004-2010 (in Chinese). Journal of Medical Pest Control 27: 843-844.

71. Song S (2011) Surveillance of diarrhea related pathogens in Sui county during 2009-2010 (in Chinese). Henan Journal of Preventive Medicine 22: 370-371.

72. Sun H, Yang X, Li Q (2011) Surveillance of Shigella in Changge in 2010 (in Chinese). Henan Journal of Preventive Medicine 22: 372-374.

73. Tian D (2011) Study of 50 pediatric accurate bacillary dysentery cases treated with Cefoperazone (in Chinese). The Medical Forum 15: 395-396.

74. Wang G (2011) Clinical characteristics and drug resistance analyses of 141 cases of S.flexneri infections in old people (in Chinese). Shanghai Journal of Preventive Medicine 23: 167-169.

75. Wang X, Wei B, Liu H (2011) Rapidly detecting enterobacteria among employees by real-time fluorescence PCR (in Chinese). China Tropical Medicine 11: 971-972.

76. Xia X, Lin Q (2011) Study of bacillary dysentery epidemics in Siyang county during 2000-2009 (in Chinese). The Medical Forum 15: 569-570.

77. Xu F, Wu L, Zhou M (2011) Integrons and antibiotic resistance of Shigella in Wenzhou: a correlativity study (in Chinese). Chinese Journal of Nosocomiology 21: 24-26.

78. Yang C (2011) Etiological and drug resistance analyses of pediatric bacillary dysentery (in Chinese). Chinese and Foreign Medical Research 9: 73-74.

1. **Small sample size**

1. Zhou H, Qin C, Zhao S (2002) Changes in etiology and drug resistance of accurate diarrhea (in Chinese). Acta Academiae Medicinae Suzhou 22: 342-346.

2. Zhan Q, Wang C, Song Z (2003) The distribution and drug resistance of Shigella in Simao region from 1992～2001 (in Chinese). China Tropical Medicine 3: 502-503.

3. Bao D, Ding K, Fan Z (2005) Etiological and drug resistance analyses of pediatric infectious enteritis (in Chinese). Modern Preventive Medicine 32: 847-848.

4. Shi Z, Yuan E, Wang Y (2005) Change of causative agents of pediatric diarrhea and drug resistance analyses (in Chinese). Journal of Medical Forum 26: 50-52.

5. Zhang X, Cai Z, Li Y (2006) Analysis on the surveillance result of diarrhea in the City of Xian in 2005 (in Chinese). Disease Surveillance 21: 470-471.

6. He A (2007) Etiological and drug resistance analyses of infectious bacillary dysentery (in Chinese). Practical Preventive Medicine 14: 222-223.

7. Li P, Hu B, Su H (2007) Flora Distribution and Analysis of Drug Resistance of Shigella in Manas County from 1992 to 2005 (in Chinese). Preventive Medicine Tribune 13: 377-378.

8. Wang Y, Zhang L, Su W (2007) Distribution and drug resistance of enteric pathogenic bacteria in some areas of Beijing (in Chinese). Journal of Preventive Medicine of Chinese People’s Liberation Army 25: 94-97.

9. Zhao H, Gu Y, Sun W (2007) Etiological and drug resistance analyses of accurate peadiatric bacillary dysentery in Dunhuang aera (in Chinese). Maternal and Child Health Care of China 22: 4266-4267.

10. Cai W, Wang H, Shen K (2008) Detection of Salmonella and Shigella from employees of public food service in Fengxian district, Shanghai (in Chinese). Shanghai Journal of Preventive Medicine 20: 119-120.

11. Luo C, Xie Y, Li H (2008) Epidemiological Analysis on Bacillary Dysentery in Fujian Province,2000-2006 (in Chinese). China Preventive Medicine 9: 712-715.

12. Zhang X, Wu S, Li H (2008) Bacteriological Surveillance of Infectious Diarrhea in 2006 in Xi’an (in Chinese). Occupation and Health 24: 352-353.

13. Xu W, Ma J, Han Q (2009) Epidemiological study of bacillary dysentery in Chaoyang district, Beijing during 2008 (in Chinese). Capital Journal of Public Health 3: 136-137.

14. Zhou W, Zhang Z, Guo L (2009) Surveillance of bacillary dysentery in Xiangcheng district, Zhangzhou during 2005-2008 (in Chinese). Strait Journal of Preventive Medicine 15: 38-39.

15. Dong L, Dang R, Liu Q (2010) Etiological study of infectious diarrhea (in Chinese). Practical Preventive Medicine 17: 2389-2390.

16. Jiang J, Li J, Wu G (2010) Shigella Isolation in Bacillary Dysentery Episodes in Outpatient Settings (in Chinese). Chinese General Practice 10: 47-49.

17. Ma S, Ge Y (2010) Species/serotype distribution, biochemical characteristics and drug resistance analyses of Shigella isolated in Tonghua during 1985-2009 (in Chinese). Chinese Journal of Ethnomedicine and Ethnopharmacy 10: 29.

18. Zhu X, Meng Z, Zhang Q (2010) Clinical Analysis of 960 Cases with Acute Bacillary Dysentery (in Chinese). Progress in Modern Biomedicine 10: 2100-2102.

19. Jin G (2011) Species distribution and drug resistance analyses of Shigella isolated from diarrhea patients in Xihu district (in Chinese). Zhejiang Journal of Preventive Medicine 23: 95-96.

20. Li G, Wang Y, Zhu Y (2011) Surveillance of diarrheal bacteria and their epidemiological characteristics in Yunan military area (in Chinese). China Tropical Medicine 11: 136-138.

21. Wang W, Qi W, Wu J (2011) Survey of Antimicrobial Susceptibility and Integron Carriage of Shigella Isolates in Tianjin (in Chinese). Tianjin Medical Journal 39: 420-423.

22. Zhang J (2011) Determination of Shigella and Drug Sensitivity Analysis in Diarrhea (in Chinese). Journal of China Traditional Chinese Medicine Information 3: 19.

23. Zhang R, Zhou HW, Cai JC, Zhang J, Chen GX, et al. (2011) Serotypes and extended-spectrum beta-lactamase types of clinical isolates of Shigella spp. from the Zhejiang province of China. Diagn Microbiol Infect Dis 69: 98-104.

24. Zhu J, Duan G, Yang H, Fan Q, Xi Y (2011) Multi-drug resistance and characteristic of integrons in Shigella spp. isolated from China. Biomed Environ Sci 24: 56-61.

1. **Inconsistent data**

1. Qiu Y, Lin Y, Zhang Q (2004) Detection of Shigella from employees of food and public service in Shenzhen (in Chinese). Practical Preventive Medicine 11: 378-379.

2. Gu G (2006) Serological and drug resistance analyses of 93 Shigella isolates (in Chinese). Chinese Journal of Hemorheology 16: 142-143.

3. Xu X, Yuan Z, Gu B (2007) Analysis of serotype spectrum and antibiotics resistance characteristic of Salmonellae and Shigella isolated from patients with diarrhea in Shanghai (in Chinese). Chinese Journal of Zoonoses 23: 706-709.

4. Wei C (2010) Etiology and characteristics of Shigella infections in Huzhu county, Qinghai province (in Chinese). China Health Care Nutrtion 19: 94-95.

5. Chen R, Gan Y, Guo X (2011) Surveillance of bacillary dysentery in Daxing district, Beijing, 2010 (in Chinese). Capital Journal of Public Health 5: 24.

6. Luo L, Wang X, Liu J (2011) Distribution and Vicissitude of Shigella in Minhang District of Shanghai City from 2003-2009 (in Chinese). Occupation and Health 27: 1732-1734.

7. Sun B, Jin Z (2011) The Feces of Diarrhea Patients Akhenaten Specimen Bacteria Culture Identification and Antimicrobial Susceptibility Test Analysis (in Chinese). National Medical Frontiers of China 6: 57-58.

8. Zhao J, Geng R, Dong X (2011) Investigation on Diagnosis Situation of Bacillary Dysentery in Fengtai District of Beijing in 2009 (in Chinese). Occupation and Health 27: 781-783.
